# An Improved Oil Palm Genome Assembly as a Valuable Resource for Crop Improvement and Comparative Genomics in the *Arecoideae* Subfamily

**DOI:** 10.3390/plants9111476

**Published:** 2020-11-03

**Authors:** Ai-Ling Ong, Chee-Keng Teh, Sean Mayes, Festo Massawe, David Ross Appleton, Harikrishna Kulaveerasingam

**Affiliations:** 1 Biotechnology & Breeding Department, Sime Darby Plantation R&D Centre, Serdang 43400, Selangor Darul Ehsan, Malaysia; teh.chee.keng@simedarbyplantation.com (C.-K.T.); david.ross.appleton@simedarbyplantation.com (D.R.A.); harikrishna.k@simedarbyplantation.com (H.K.); 2 School of Biosciences, University of Nottingham, Sutton Bonington Campus, Leicestershire LE12 5RD, UK; sean.mayes@nottingham.ac.uk; 3 School of Biosciences, University of Nottingham Malaysia, Semenyih 43500, Selangor Darul Ehsan, Malaysia; festo.massawe@nottingham.edu.my

**Keywords:** *Elaeis guineensis*, high-density linkage maps, physical maps, comparative genomics, genome-enabled breeding, *Arecoideae*, pseudomolecules

## Abstract

Oil palm (*Elaeis guineensis* Jacq.) is the most traded crop among the economically important palm species. Here, we report an extended version genome of *E. guineensis* that is 1.2 Gb in length, an improvement of the physical genome coverage to 79% from the previous 43%. The improvement was made by assigning an additional 1968 originally unplaced scaffolds that were available publicly into the physical genome. By integrating three ultra-dense linkage maps and using them to place genomic scaffolds, the 16 pseudomolecules were extended. As we show, the improved genome has enhanced the mapping resolution for genome-wide association studies (GWAS) and permitted further identification of candidate genes/protein-coding regions (CDSs) and any non-coding RNA that may be associated with them for further studies. We then employed the new physical map in a comparative genomics study against two other agriculturally and economically important palm species—date palm (*Phoenix dactylifera* L.) and coconut palm (*Cocos nucifera* L.)—confirming the high level of conserved synteny among these palm species. We also used the improved oil palm genome assembly version as a palm genome reference to extend the date palm physical map. The improved genome of oil palm will enable molecular breeding approaches to expedite crop improvement, especially in the largest subfamily of *Arecoideae*, which consists of 107 species belonging to *Arecaceae.*

## 1. Introduction

The *Arecaceae* (formerly known as *Palmeae*) family of the monocot order *Arecales* is a plant family hosting perennial palm species that mostly grow across the equatorial belt. The family is made of 181 genera with 2600 species [1]. Among them, the genus *Elaeis* (oil palm) and genus *Cocos* (coconut) together are the world’s largest source of oils and fats (about 35%), while date palm plays a major role in food security and agricultural production in arid and fragile ecosystems worldwide. 

Two species of *Elaeis* including *E. guineensis* Jacq. and *E. oleifera* (HBK) Cortes originating from West Africa and Latin America, respectively, produce edible oil from fruit mesocarp and kernel. However, only the African oil palm is typically commercially planted due to its superior oil yield with an average oil yield in Malaysia of 3.5 tons hectare^−1^ year^−1^, compared to only 0.4 tons hectare^−1^ year^−1^ produced by its South American counterpart [2]. The average oil yield of commercial oil palm is also roughly 8 to 10 times higher than other major temperate oil crops [3], making it the most efficient oil crop in the world. However, with increasing demand for fats and oils from a growing population, yield improvement is essential to meet food requirements at the same time as halting deforestation. Crop improvement through breeding and genomics is the basis for increasing yield and therefore productivity without the use of more land.

Although coconut (*C. nucifera* L.) is not as productive as oil palm, it generates more products such as copra, coir, and timber to serve traditional markets, particularly in Asia. Nut yield in Asia is approximately 5.5 tons hectare^−1^ year^−1^, while the copra yield ranges from 10 to 20 percent of the nut weight, equivalent to around 1 ton hectare^−1^ year^−1^ [4]. The recent rise in demand for coconut products has resulted from coconut water and virgin coconut oil being promoted as an energy drink and health supplement. The market size of coconut products was reported to be USD 11.5 billion in 2018 and is anticipated to reach USD 31.1 billion by 2026 [5]. Another related palm species, the date palm (*Phoenix dactylifera* L.) does not produce oil but stores mostly carbohydrate, which is converted to sugar in its fruit mesocarp [6]. Hence, the crop has been domesticated in the Middle East and Indus Valley as a major staple food, and the earliest cultivation can be traced back to 3700 BC [7]. Date palm thrives in some of the most arid environments on earth and is able to create a local microclimate, which also allows other crops to be grown [8]. Similarly, higher global demand for inherent medicinal, nutritional, and health advantages of date palm products has boosted the market value of the date palm industry from USD 9.2 billion in 2014 to USD 13 billion in 2018 [9]. 

Within the *Arecaceae*, there are many other palms that could make a significant contribution to economic development, food, and nutritional security and provide ecosystem services for sustainable cultivation, including sago palm, nipa palm, peach palm, etc. However, it is unlikely that companies will be willing to make major investment in developing genetic improvement programs without being sure that there will be sizable returns. To date, oil palm receives the most research attention due to its significant role in the world economy, although coconut and date palm have seen steep increases in research focus with their recent emergence. In general, genomic research is centered on the development of marker-assisted selection (MAS) for expediting breeding selection for these outcrossing perennial palms that typically require more than a decade to complete a breeding cycle [10,11,12,13,14]. In the 90s, the first restriction fragment length polymorphism (RFLP)-based linkage map of oil palm [10] was reported and subsequently deployed for mapping quantitative trait loci (QTL) responsible for fruit characteristics and vegetative traits [15]. The effort was continued and expanded using various DNA marker systems: random amplified polymorphic DNA (RAPD), amplified fragment length polymorphism (AFLP), diversity arrays technology (DArT), simple sequence repeat (SSR), and single nucleotide polymorphism (SNP) [10,16,17,18,19,20,21]. The reported QTL, however, were only applicable to a few populations closely related to the mapping populations. In oil palm, the Deli *dura* that produces a thick kernel shell, high bunch number, and high mesocarp oil content and is widely used in different programs and subsequently has been developed in breeding populations to different sub-populations, such as *Johore Labis, Ulu Remis*, and *Gunung Melayu.*

Mapping resolution, especially for complex traits (e.g., oil yield and disease tolerance), is limited by insufficient recombination and a lack of statistical power within a small bi-parental family, which usually consists of only 16 to 96 field-planted palms in which traits are recorded in breeding programs. To improve mapping resolution, research efforts gradually shifted towards genome-wide association studies (GWAS) to access total meiotic recombination accumulated in a large admixed population. Nevertheless, the mapping method was of limited power until the reference *E. guineensis* genome (AVROS *pisifera*), namely P5-build, was sequenced and made publicly available in 2013 [22]. We then reported the first GWAS for mesocarp oil content based on 2045 *tenera* oil palms [23] using a high-density OP200K SNP array [24]. However, only 60% of the linked SNPs were located across 16 chromosomes (or pseudomolecules) of the reference oil palm genome, with the P5-build (2n = 32), leaving room for improvement. The same limitations on GWAS were also observed in the date palm [25]. 

A good genome assembly does not only rely on assembly of contigs into scaffolds but also the placement and contiguity of scaffolds on chromosomes to form the physical map. To summarize the latest status of assembled genomes of oil palm, coconut, and date palm (as available in National Centre for Biotechnology Information; NCBI): 1535.18 Mb of P5-build oil palm genome first achieved a chromosome coverage level with 43% of scaffolds anchored [22], although no improvement has been published since 2013. On the other hand, the 2202.46 Mb of assembled coconut genome and the 772.32 Mb of assembled date palm genome have recently surpassed the oil palm genome, with 46% [26] and 50% [25] of scaffolds anchored to the chromosomes. The genome size of oil palm is estimated at about 1.8 Gb [27,28]. We previously reported on the possibility of further extending the physical map of the current P5-build reference genome using one ultra-dense linkage map of a commercial Deli *dura* × AVROS *pisifera* population, and the total length of additional scaffolds anchored in our previous study has improved by 311 Mb [29]. Hence, our aim in this study was to maximize the improvement of the oil palm genome assembly by integrating multiple linkage maps from various origins. Here, we also report how the improved oil palm assembly, namely using Physical Map version 6 (PMv6), can serve as an important reference for comparative genomic studies among coconut and date palms, with expansion to other *Arecaceae* members in the future.

## 2. Results

### 2.1. Construction of Multiple High-Density Oil Palm Linkage Maps

Three bi-parental populations, including the reported commercial Deli *dura* × AVROS *pisifera* (295 *tenera* palms) [29], semi-wild Deli *dura* × Nigerian *dura* (112 *dura* palms), and *Johore Labis dura* × *Johore Labis dura* (214 *dura* palms) were used to construct individual linkage maps. We obtained 17 to 24 linkage groups (LGs; Table 1) with one to six chromosomes being represented by two or three linkage groups representing the expected 16 major LGs for total oil palm chromosomes number. The *Johore Labis dura* population showed the lowest number of linked SNPs (6920 loci; due to the use of refined SNP chip of 20K loci), while the highest number of linked SNPs (32,650 loci) was observed for the Deli *dura* × Nigerian *dura* population. The Deli *dura* × AVROS *pisifera* map remained the densest with a mean inter-marker distance of 0.04 cM, as reported [29], compared to the sparsest *Johore Labis* map (0.18 cM). In this study, a total of 11,421 common SNPs was identified and adopted for integration of the multiple linkage maps.

### 2.2. Improvement of the Oil Palm Physical Genome Assembly by Integration of Multiple Oil Palm Linkage Maps

**Figure 1:**
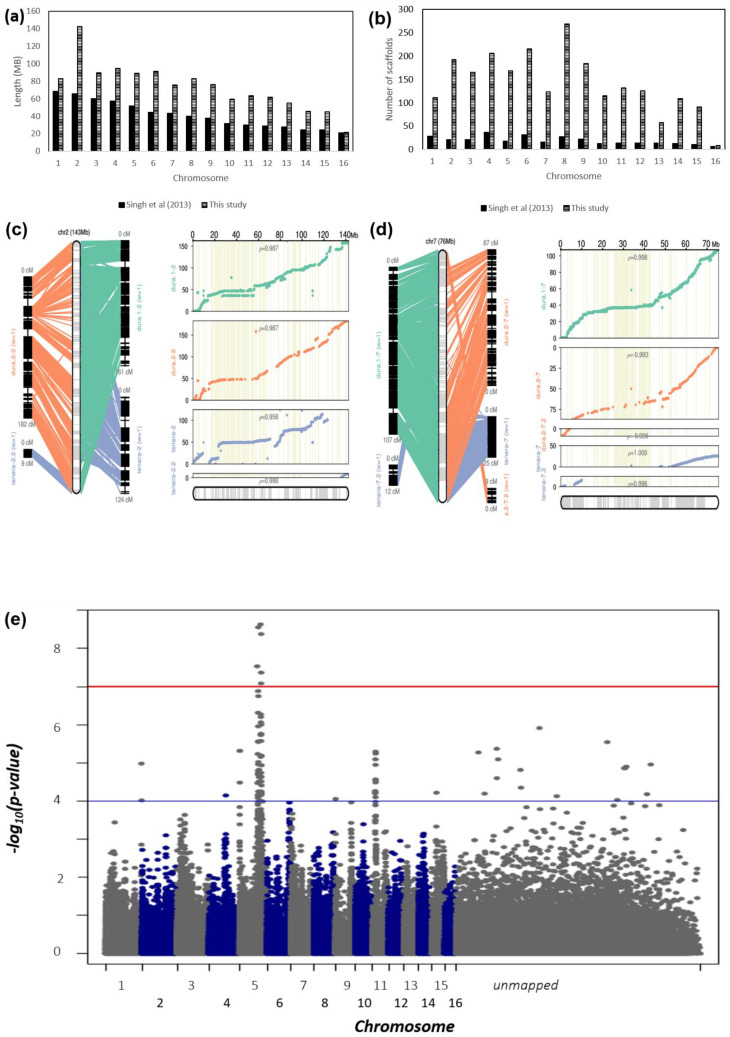

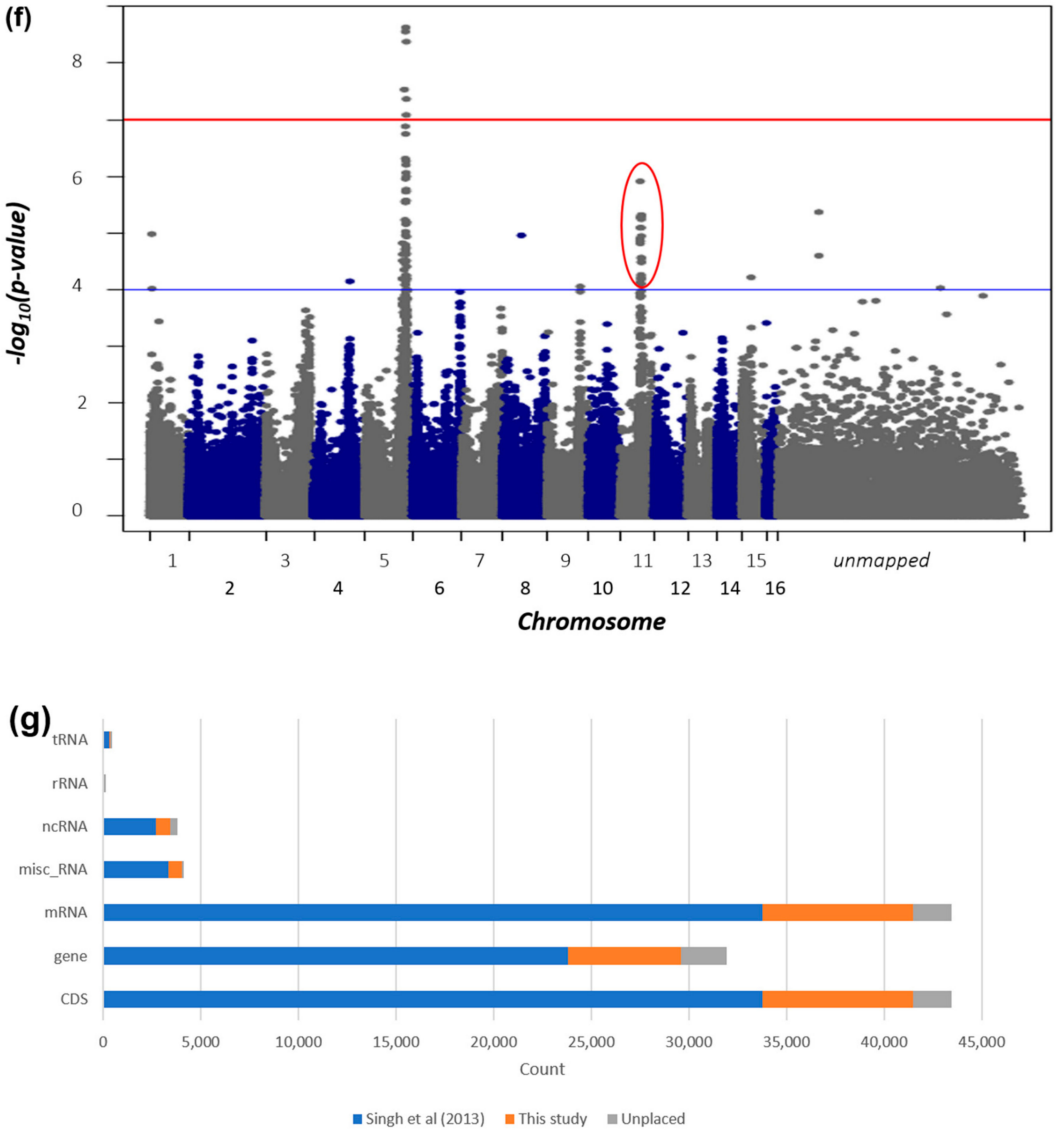
Refined assembly of oil palm genome (PMv6). (**a**) Chromosome length (Mb) comparison between P5-build, Singh et al., (2013), and PMv6 (this study). (**b**) Number of scaffolds from previous physical map [22]) and the improved physical map developed in this study. (**c**) Buildup of PMv6 by scaffold anchoring using multiple linkage maps; left panel represents the central combined linkage map from three mapping populations, and the right panel shows the correlation plots of each populations’ genetic versus physical order; presented in the order of Deli *dura* × Nigerian *dura* (*dura* 1—green), *Johore Labis dura* (*dura* 2—orange), and Deli *dura* × AVROS *pisifera* (*tenera*—blue) for Chromosome 2 and (**d**) Chromosome 7 as examples. (**e**) Genome-wide association studies (GWAS) Manhattan plot for a commercial oil palm population for one of the oil yield trait [23] for the genome published by P5-build. (**f**) GWAS Manhattan plot for the same commercial oil palm population for one of oil yield trait after assembly improvement in this study, circle in red represent newly assigned and significant SNPs. (**g**) Statistics of genomic features improvement after placement of scaffolds into chromosomes for P5-build and this study.

Linkage-directed genome assembly improvement not only anchored previously unplaced scaffolds to the pseudomolecules but also split and reassigned a range of the scaffolds flanked by N bases in the published physical genome, P5-build [22]. The newly assembled genome, termed PMv6 with an N50 of 83.1 Mb, improved the scaffold assignment to oil palm pseudomolecules from 43% to 77% (1.2 Gb, Figure 1). On average, each pseudomolecule in the PMv6 genome was assembled using 142 scaffolds with an average length of 73.7 Mb, whereas only 19 scaffolds giving an average length of 41.1 Mb were reported in Singh et al., 2013 [22]. Chromosome 2 was also extended by 1.2-fold and now is the largest pseudomolecule, accounting for 12% of the total PMv6 assembly length (Figure 1a,b).

In Figure 1c,d, we illustrate how the scaffolds were anchored to Chromosome 2 and 7 based on the integrated linkage maps, and how they were validated by correlation between linkage and physical positions (Figure 1c,d). The results show that assembly improvement using the commercial Deli *dura* × AVROS *pisifera* map [29] (blue) was only limited to one end of the pseudomolecule, especially on Chromosome 7. Nevertheless, the shortcomings were solved when integrating the semi-wild Deli *dura* × Nigerian *dura* map (green) and the *Johore Labis dura* map (orange). The sigmoidal curves of Chromosome 2 and 7 clearly distinguishing both telomere regions from centromeric regions where limited recombination events were observed. The correlation between linkage and physical positions reached more than 0.90 in each mapping family, and the collinearity between each linkage maps can be observed with consistence marker orderings within each LG. Similar results were observed for the remaining chromosomes and are shown in Supplementary Figure S1. The details of total SNPs residing within genomic scaffolds used to build PMv6 genome assembly can be found in Supplementary Table S1. Using the correlation outputs, the genome-wide recombination rate was estimated to be 1.15 cM/Mb, which is about half of the previously reported estimation based on the P5-build genome [29] (Table 1). This investigation may infer overestimation of the recombination rate in our previous estimation, mainly due to the length extension of the PMv6 genome assembly reported here. Understanding recombination differences has distinct implications for population structure, gene evolution, and genetic improvement [30]. 

The improved PMv6 genome was used to evaluate the improvement of mapping resolution of the previously reported GWAS for mesocarp oil content [23], reducing the unmapped SNPs from 40% (37,003) (Figure 1e) to only 13% (12,175) (Figure 1f). The major QTLs still remain at Chromosome 5 and Chromosome 11, but the significance level on Chromosome 11 was increased to a –log *p*-value = 5.9 from 5.2 and more SNPs surpassed genome-wide -log *p*-value cutoff at 4.0, due to SNPs newly assigned to Chromosome 11, improving trait location. From the perspective of genomic features and gene annotations, the PMv6 genome assembly now includes 5769 genes, 7705 protein-coding regions (CDS), and 1563 non-coding RNA, which could not be placed into the P5-build genome (Figure 1g). Interestingly, among the non-coding RNA, 48% of total predicted ribosomal RNA (rRNA) were mapped for the first time in PMv6. 

### 2.3. Comparative Genomic Analysis of Three Palm Species

**Figure 2:**
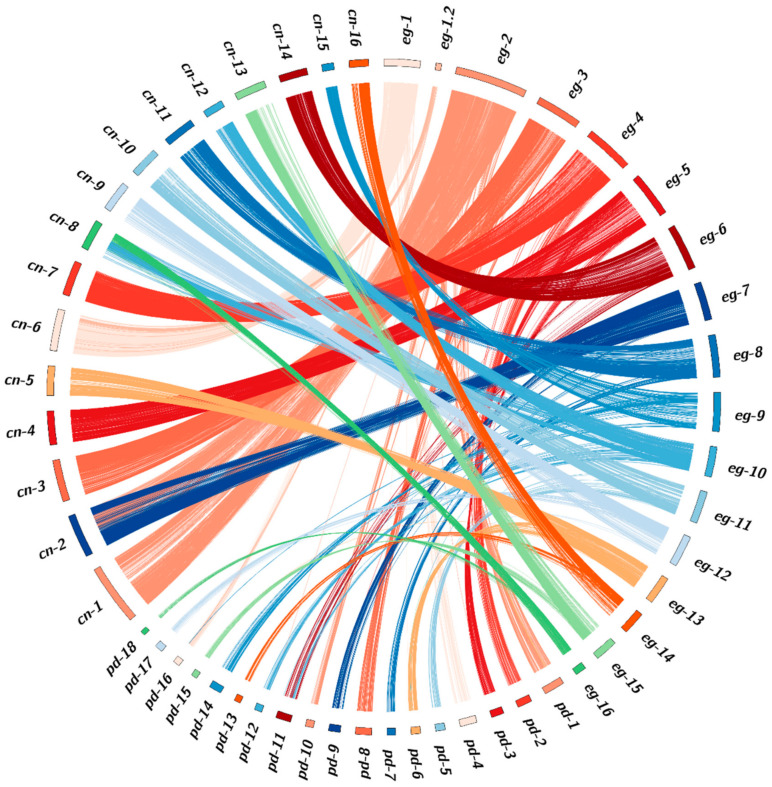
Synteny analysis including three palm species: oil palm (eg), coconut (cn), and date palm (pd). Chromosome pairs with strong syntenic relations were linked by the same ribbon color.

The improved oil palm genome, PMv6 was subsequently used for genome comparison with coconut [26] and date palm [25]. The results confirmed a high level of syntenic block conservation between the oil palm and the other two palm species (Figure 2). For coconut, the 16 homologous chromosome groups (2n = 32) appeared to be highly coherent with the oil palm genome, but we observed possible fusion and fission event differences on oil palm-based Chromosome 2 (eg-2) and 10 (eg-10), respectively. Compared to coconut, a 30 Mb fragment of eg-2 fused with eg-7 accounts for coconut-based Chromosome 2 (cn-2), suggesting a fission/fusion event since the separate evolution of the two species. In a similar comparative way, a 20 Mb fragment of eg-10 has fused with eg-16 to form cn-8. 

In general, the genome conserved synteny between date palm and oil palm was more complicated. By referring to Ziwen et al. (2015), oil palm and coconut were estimated to have diverged from date palm approximately 62.5 million years ago [31]. Chromosome fusion/fission events between date palm-based Chromosome 4 (pd-4) and pd-16 and between pd-1 and pd-10 may have led to formation eg-1 and eg-2 in the oil palm genome, respectively. This possibly explains why date palm (2n = 36) has two additional homologous chromosome groups compared to oil palm. In addition, pd-11 compared to oil palm has two chromosome fragments with one of them fused with pd-12 to form eg-10 and the remaining pd-11 fragment being maintained as eg-6 in the oil palm genome. 

**Figure 3:**
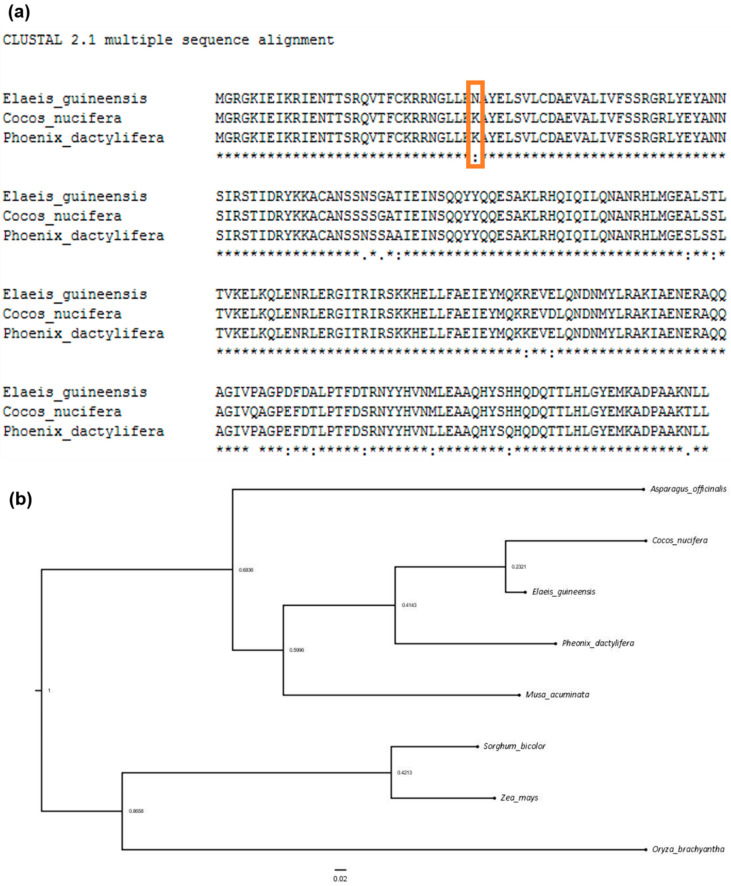
High conservation of genes among palm species with minimal amino acid changes. (**a**) Alignments of *SHELL* gene encoding for the layer of shell surrounding the kernel for oil palm and the orthologues from coconut and date palm. (**b**) Phenogram tree for *LOG* gene orthologues, which play a role in sex determination for date palms; orthologues of the gene among palms and other monocots outgroup taxa were included.

We then further compared the *SHELL* gene, which is a homologue of *SEEDSTICK* (NCBI Gene ID: LOC105034563)—a type II MADS-box transcription factor reported in oil palm (at 3.05 Mb of eg-2) with the orthologs of coconut (at 116 Mb of cn-1) and date palm (at 38 Mb of pd-1). In the oil palm genome, the *pisifera* allele (*sh^AVROS^*) results in an amino acid change from lysine to asparagine, causing the absence of shell surrounding the fruit kernel observed *pisifera* fruit [32] (Figure 3a). The analysis was also extended to other candidate genes including Cytochrome P450 (*CYP703*) (LOC105059962), glycerol-3-phosphate acyltransferase 3-like (*GPAT3*) (LOC105059961), Cytidine deaminase-like (*CYT DA*) (LOC105059743), and Lonely-Guy (*LOG*) (LOC105055182) reported to be involved in sex determination of date palm [33]. All the candidate genes (except *LOG*, located at 10.86 Mb of eg-12) were located at 44–45 Mb of eg-10 in the oil palm genome but have yet to have the scaffolds containing them placed in the date palm genome. 

In Figure 3b, a phenogram based on the sex-determination-related *LOG* genes revealed that coconut has the shortest evolutionary distance to oil palm followed by date palm, among other monocots outgroup taxa (Figure 3b).

### 2.4. Oil-Palm-Guided Improvement of the Date Palm Genome

**Figure 4:**
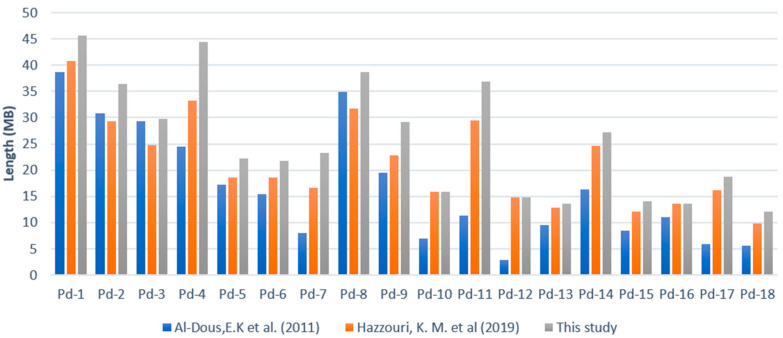
Oil-palm-directed date palm genome assembly improvement by length (Mb) across 18 pseudomolecules. Anchoring of scaffolds was performed on the two published assemblies of date palm genome by Al-Dous et al. (2011) [34] and later by Hazzouri et al. (2019) [25].

Due to the high levels of collinearity of gene order and genomic sequences between the palm species, the PMv6 genome was adopted as a reference to improve the published genome assemblies of date palm. We started with the earlier version, namely PDK_30 (GenBank assembly accession: GCA 000181215.2) [34]. Genomic scaffolds from date palm were tiled following oil palm ordering for each pair of syntenic chromosomes, then the joined scaffolds were split if required following the published date palm genetic map [35]. This resulted in anchoring 78% of scaffolds to the physical map from the total length of the full assembly (381.5 Mb). The next test was performed on the more recent assembly of date palm genome (GCA_009389715.1) [25] using the same approach. The second improvement was not as apparent as the first version but did result in a total length of 458 Mb, equivalent to an extra 10% of total scaffold length anchored to chromosomes, compared to the published assembly. The placement of scaffolds in each of the date palm chromosomes extended their total length except for pd-10 and pd-16 (Figure 4). The assembly improvement for both versions of the date palm genome were further compared using date palm genetic markers [35] located within each assembly. An average correlation of 0.80 was observed for the 18 chromosomes when comparing physical location of the genetic markers between both improved assemblies, ranging from the lowest on pd-16 (*r* = 0.38) to the highest on pd-4 and pd-15 (equally *r* = 0.98) (Figure S2).

## 3. Discussion

In our recent publication, we demonstrated an extension of 311 Mb of the published oil palm pseudomolecules (P5-Build [22]) using a linkage map of a commercial Deli *dura* × AVROS *pisifera* family (295 palms) [29]. In this study, we constructed two denser linkage maps from a semi-wild Deli *dura* × Nigerian *dura* population (112 palms) and *Johore Labis dura* population (214 palms). The Deli *dura* origin is derived from four palms planted at Bogor Botanical Garden in 1848 [36] and exhibits a thick kernel shell, high bunch number, and high mesocarp oil content. It has become the most important genetic resource for major breeding programs in Southeast Asia. Separate breeding programs using Deli *dura* have led to sub-populations, such as *Ulu Remis* and *Johore Labis* [37]. Breeding history and selection have likely resulted in the lowest number of linked SNPs and the lowest mapping density being observed in the pure *Johore Labis dura* population (Table 1). To avoid inbreeding depression, Sime Darby Plantation R&D also introgressed the Deli *dura* with a Nigerian *dura* to widen the genetic base, reflected by the higher mapping density and number of linked SNPs observed. 

By integrating the three linkage maps, a total of 11,421 family-transferable/common SNPs were used to anchor 77% of the assembled 1.5 Gb genome, in order to generate the new oil palm physical map, PMv6 (Figure 1), compared to 43% in the previously published P5-build physical map. Using Chromosome 2 and 7 as examples, the quality of scaffold anchoring based on the integrated linkage maps can be visualized and was validated through a correlation between genetic and physical positions, allowing the recombination landscape throughout each chromosome to be determined (Figure 1c,d). Species with larger genomes often have higher levels of recombination at the telomeres and lower recombination in the heterochromatic and centromeric regions despite genes being present within transposable element (TE) [38,39] clusters, as reported in wheat [40,41]. In such a scenario, a sigmoidal curve of the recombination rate should be expected, but this was not clearly observed in both chromosomes when assembled from the commercial Deli *dura* × AVROS *pisifera* map (blue), suggesting only a partial improvement. However, the integration of the semi-wild Deli *dura* × Nigerian *dura* map (green) and the *Johore Labis dura* map (orange) enabled extension of both chromosomes with a sigmoidal curve of recombination from telomere-to-telomere, although whether this represents the full chromosome needs further validation, through techniques such as Fluorescence in situ hybridization (FISH) technique. 

The PMv6 genome enabled an increase in the mapping resolution of the reported GWAS for mesocarp oil content [23] by locating an additional 65% (24,828) previously unplaced SNPs (Figure 1f). Among the total SNP markers mapped in PMv6 assembly, there are 32,552 SNPs located within genic/non-coding RNA regions, equivalent to 25% of the total SNP assigned to scaffolds/genome sequence. Increased marker density allows better capture of linkage disequilibrium between origins and render GWAS more powerful. Hence, PMv6 enables a more complete set of annotated genes and non-coding RNA to be located that are potentially responsible for phenotypic changes residing within QTL to be identified after conducting GWAS.

In general, most of the chromosomes between the oil palm, coconut, and date palm genomes are highly syntenic (Figure 2). They also share agriculturally desirable traits, such as tall or short varieties in coconut palm, which would be useful in oil palm and date palm. It would be possible to use the causative gene from coconut to screen for allelic differences in the gene orthologues in other species. For instance, the *SHELL* gene in the oil palm genome (eg-2) was found to be highly conserved with the genomic sequence orthologs in coconut (cn-1) and date palm (pd-1). Only the oil palm genome sequence carries *sh^AVROS^* variants causing the lysine-to-asparagine amino acid change, which results in disruption of DNA binding within the MADS-box domain [32], thus no shell is found in this *pisifera* oil palm. Indeed, two more fruit forms including thick-shelled dura (*sh^Deli^/sh^Deli^* as wild-type) and thin-shelled *tenera* (*sh^Deli^ /sh^AVROS^*) also naturally exist because of haploinsufficiency effect on the *SHELL* gene [32,42]. The *SHELL* variants are extremely important for oil palm breeding, because crossing between dura and *pisifera* (usually female sterile) produce *tenera* with fertility restored and 30% more oil than the dura [43,44]. Unlike oil palm, shell-less fruits in coconut and date palm have not yet been reported. The shell layer may be essential to coconut and date palm for maintaining their endosperm moisture content to allow seed germination, especially in arid habitats. Consequently, the mutation probably has been naturally selected out. Nevertheless, we still identified a strong QTL for shell thickness of *tenera* oil palms located within 2.5 Mb to 3.9 Mb sharing the same region with the *SHELL* gene on eg-2 [24], even when the later was fixed. Hence, the results obtained from oil palm research could lead to the same region in coconut palm being screened for the orthologs responsible for shell variation in coconut within cn-1 and eventually deployed in MAS for selecting thicker- or thinner-shelled coconuts. Coconut shell normally occupies 15% of the total weight of a fruit and approximately 9 million tons of coconut shell is globally produced as an agricultural waste every year [45]. Nevertheless, the waste can be turned into activated charcoal and commands a premium [46,47]. 

Oil palm and coconut are estimated to have diverged from date palm around 62.5 million year ago [31]. Fission or fusion of two chromosomes into one or vice versa is known in many lineages [48]; this event is observed in this study too, whereby date palm has 18 pairs of chromosomes, while oil palm and coconut have only 16 pairs. Torres et al. found that the sex-determination-related genes are now located at 44–45 MB of eg-10 [33], where fusion between pd-11 and pd-12 probably occurred, comparative to oil palm. This also suggests that eg-10 contains this cluster of key genes for inflorescence gender and that the split into two chromosomes in the date palm may be an important event leading eventually to a diecious palm. The finding is supported by a shorter evolutionary distance of LOG gene (suppression on female inflorescence) between oil palm and coconut, compared to date palm (Figure 3b). Of the three palms, date palm is the only diecious palm. The cycle of male and female inflorescences of monoecious oil palm is mainly affected by environmental and genetic factors. Water stress and excessive frond pruning lead to high induction of male inflorescences and abortion of both sexes when the stress becomes extreme [49]. Understanding the genetic control underlying sex determination in oil palm can provide a new avenue to address abiotic stress tolerance in oil palm. Understanding the basis of inflorescence sex determination may allow breeding for specific environments, to optimize sex ratio to the target environment, especially in the context of global climate change and of plantations in drought-prone areas. In terms of aptitude for branching, date palm and sago palm can also propagate vegetatively by producing suckers, which would be an extremely valuable trait in oil palm, where this currently does not happen. 

Only 19% of the scaffolds were anchored based on a linkage map of the Khalas cultivar to the first date palm assembled genome, PDK_30 [34,35]. Subsequently, the genome has recently been greatly improved to 50% anchored scaffolds of the expected 772.32 Mb genome size using third generation sequencing PacBio long reads by Hazzouri et al. [25]. In the present study, we have shown that the new oil-palm-guided date palm assembly is still able to anchor a further 10% of scaffolds, and the oil-palm-directed assembly is highly consistent with the latest date palm assembly. Hence, a full utilization of available linkage maps, especially with high mapping density should be incorporated in genome improvement. The oil palm PMv6 genome assembly can serve as a reference to date palm and coconut, and to other palm members in the future. Moreover, based on the taxonomic distances between oil palm and date palm, we would expect similar results from any palm species with a divergence time less than 62.5 million years [31] ago within *Arecoideae*, a subfamily of *Arecaceae* that consists of 107 species [50,51], using the oil palm PMv6 genome as a reference. Improved genome sequences can in turn lead to crop improvement strategies for these important food, feed, and fuel sources that will ultimately lead to the higher productivity required for agriculture to meet growing demands in the years to come without the use of more forest land.

## 4. Materials and Methods

### 4.1. Mapping Populations and DNA Preparations

Three mapping populations from oil palm breeding populations of restricted origins (BPROs) including the 295 commercial *tenera* palms derived from a Deli *dura* × AVROS *pisifera* family (278 × TT41/4), previously described in Ong et al. [29], were used. The other two populations were maintained as female parental breeding sources, namely the Deli *dura* × Nigerian *dura* and *Johore Labis dura* × *Johore Labis dura* with population sizes of 112 and 214 individuals, respectively. Leaf samples were collected from each palm planted in different breeding trials maintained by Sime Darby Plantation Research and Development Centre, Sime Darby Plantation Sdn Bhd, Malaysia. Fresh leaf tissue was sampled from the third frond of each palm. Samples of genomic DNA were isolated from 0.1 g of the leaf tissue using the DNAeasy Plant Mini Kit (Qiagen, Germany). The DNA concentration and purity were quantified on a 0.8% agarose gel using known standards.

### 4.2. SNPs Identification and Genotyping

The extracted genomic DNA (25 ng/μL) samples from Deli *dura* × Nigerian *dura* and Deli *dura* × AVROS *pisifera* were genotyped using the 200K SNPs included on the published OP200K Infinium array (Illumina, USA) [24], and the population *Johore Labis dura* × *Johore Labis dura* was genotyped using another 20K SNP panel, selected as a subset of the 200K panel on the Infinium iScan platform according to the manufacturer’s recommendations. Raw genotyping data were analyzed with GenomeStudio version 20011.1 with genotyping module version 1.8.4. Using a GenCall score cutoff of 0.15, auto-clustering of the SNPs was done. The SNP clustering was confirmed manually by visual inspection. The SNP calls were exported into the PLINK program for minor allelic frequency (MAF) cutoff at 0.01 [52]. Polymorphic SNPs were identified where at least one of the parents was heterozygous. Subsequently, the polymorphic SNPs were dropped where the call rate <95% or where there was significant segregation distortion, using a false discovery rate (FDR)-corrected method with an effective cutoff at *p*-value < 0.05.

### 4.3. Construction of Linkage Maps

All linkage maps were constructed using Lep-MAP3 [53,54] for large SNP datasets, especially developed for outbred families such as oil palm with an underlying maximum likelihood (ML) method. Filtering module was used for marker quality checking, followed by running the SeparateChromosomes module for binning the assayed markers into linkage groups with optimized LOD values ranging from 5 to 15 with intervals of 5. The JoinSingles module assigned singular markers to existing linkage groups to maximize the map ability of the total input marker set. Lastly, the OrderMarkers module ordered the binned markers according to the Kosambi mapping function for conversion of recombination frequencies into map distances (in unit centiMorgan, cM). The option “sexAveraged = 1” was selected along with OrderMarkers to join the maps of both parents. 

### 4.4. Anchoring of Scaffolds to Linkage Groups

Linked SNP markers (from OP200K) with flanking sequences of 60mers located in the oil palm scaffolds (placed and unplaced in pseudomolecules) were identified using BLASTN [55] with e-value cutoff at 1 × 10^−150^ and sequence identity at least of 95%. High-density linkage maps built from the three mapping populations were merged using ALLMAPS assembly module, together with equal weighting among the linkage maps [56]. Scaffolds were then ordered and orientated according to the merged and weighted linkage maps, thus resulting in the new genome content. The same parameters have been applied to date palm SNP markers with flanking sequences of 100mers to identify the locations of the markers in two of the date palm genome versions.

### 4.5. Genome and Gene Comparisons

Palm genomic sequences were downloaded from NCBI. Alignments of the coconut [26] and date palm [57] genome against the current oil palm genome (PMv6) was done using Nucmer, which is a module developed in Mummer [58,59], an ultrafast sequences aligner, made for genome alignments. The syntenic relationship between both oil palm and coconut chromosomal alignments were visualized using the CIRCOS software [60]. Chromosome pairs with strong syntenic relations between oil palm, coconut, and date palm were linked by same ribbon colors. Candidate genes of interest were also aligned using multiple sequence alignment tool, clustalw [61,62] via translated protein and orthologs of other closely related monocots as outgroup: asparagus (*Asparagus officinalis*), banana (*Musa acuminata*), sorghum (*Sorghum bicolor*), corn (*Zea mays*), and turf (*Oryza brachyantha*) protein sequences were identified from NCBI. The phenogram was build using neighbor-joining (NJ) with 1000 bootstrap iterations and visualized using a phylogenetic tree viewer, namely Figtree (v1.4.4) [63].

### 4.6. Reconstruction of Date Palm Pseudomolecules 

Mummer (Nucmer) output from the step above tiled the date palm’s query scaffolds according to the sequence order in the newly extended oil palm pseudochromosomes. Those scaffolds were then ordered and orientated into GenBank agp formats, and the pseudomolecules were built using the agp module in ALLMAPS. SNP markers obtained from the published date palm genetic maps [35] were aligned to the new pseudomolecules using the same BLASTN criteria, as above. The integration of SNP marker locations in the genetic map against physical map location was plotted using ALLMAPs path module. Chimeric contigs, which contained ambiguous markers that mapped to more than two linkage groups, were then processed using the split module. Ambiguous short contigs were excluded during the second round of rebuild of pseudomolecules by running the ALLMAPs path module again. 

## Figures and Tables

**Table 1:** Statistics of linkage maps developed for each oil palm population.

Population	Number of Linked Markers	Number of Linkage Groups (LGs)	Length of Linkage Map (cM)	Marker Interval (cM)	Genome-Wide Recombination Rate of P5-Build (cM/Mb)	Genome-Wide Recombination Rate of PMv6 (cM/Mb)
Deli *dura* × AVROS *pisifera* *	27,890	19	1151.70	0.04	1.75	0.98
Deli *dura* × Nigerian *dura*	32,650	17	1646.95	0.05	2.50	1.40
*Johore Labis dura*	6920	24	1268.26	0.18	1.93	1.08

* previously reported [29].

## Supplementary Materials

The following are available online at https://www.mdpi.com/2223-7747/9/11/1476/s1, Figure S1: Integration of genetic with physical maps for oil palm, Chromosomes 1 to 16; Figure S2: Correlation plots for locations of DNA marker located within date palm chromosome (pd-15) for genome assembly improvement versions by Al-Dous et al. (2011) and Hazzouri et al. (2019); Table S1: Summary for consensus map generated from multiple linkage maps and statistics for SNP markers and scaffolds used to build genome assembly for PMv6. The assembled genome has been uploaded in NCBI with submission number: SUB7516020/ BioProject: PRJNA636092.
