# Supplementary material for: An Improved Oil Palm Genome Assembly as a Valuable Resource for Crop Improvement and Comparative Genomics in the Arecoideae Subfamily
Source: Plants (Basel). 2020 Nov 3;9(11):1476. doi: 10.3390/plants9111476 (PMC7692215; doi:10.3390/plants9111476)
Supplement: Supplementary file 1 [file plants-09-01476-s001.pdf]

An Improved Oil Palm Genome Assembly as a Valuable Resource for Crop Improvement and Comparative Genomics in the *Arecoideae* Subfamily

Ai-Ling Ong [1,2,*], Chee-Keng Teh [1,2], Sean Mayes [2], Festo Massawe [3], David Ross Appleton [1] and Harikrishna Kulaveerasingam [1]

[1] Biotechnology & Breeding Department, Sime Darby Plantation R&D Centre, Serdang 43400, Selangor State, Malaysia; teh.chee.keng@simedarbyplantation.com (C-K.T); david.ross.appleton@simedarbyplantation.com (D.R.A.); harikrishna.k@simedarbyplantation.com (H.K)

[2] School of Biosciences, University of Nottingham, Sutton Bonington Campus, Leicestershire, LE12 5RD, UK; sean.mayes@nottingham.ac.uk

[3] School of Biosciences, University of Nottingham Malaysia, Semenyih 43500, Selangor State, Malaysia; festo.massawe@nottingham.edu.my

* Correspondence: ong.ailing.sdtc@simedarbyplantation.com

Figure S1. Integration of genetic with physical maps for oil palm, Chromosome 1 to 16.

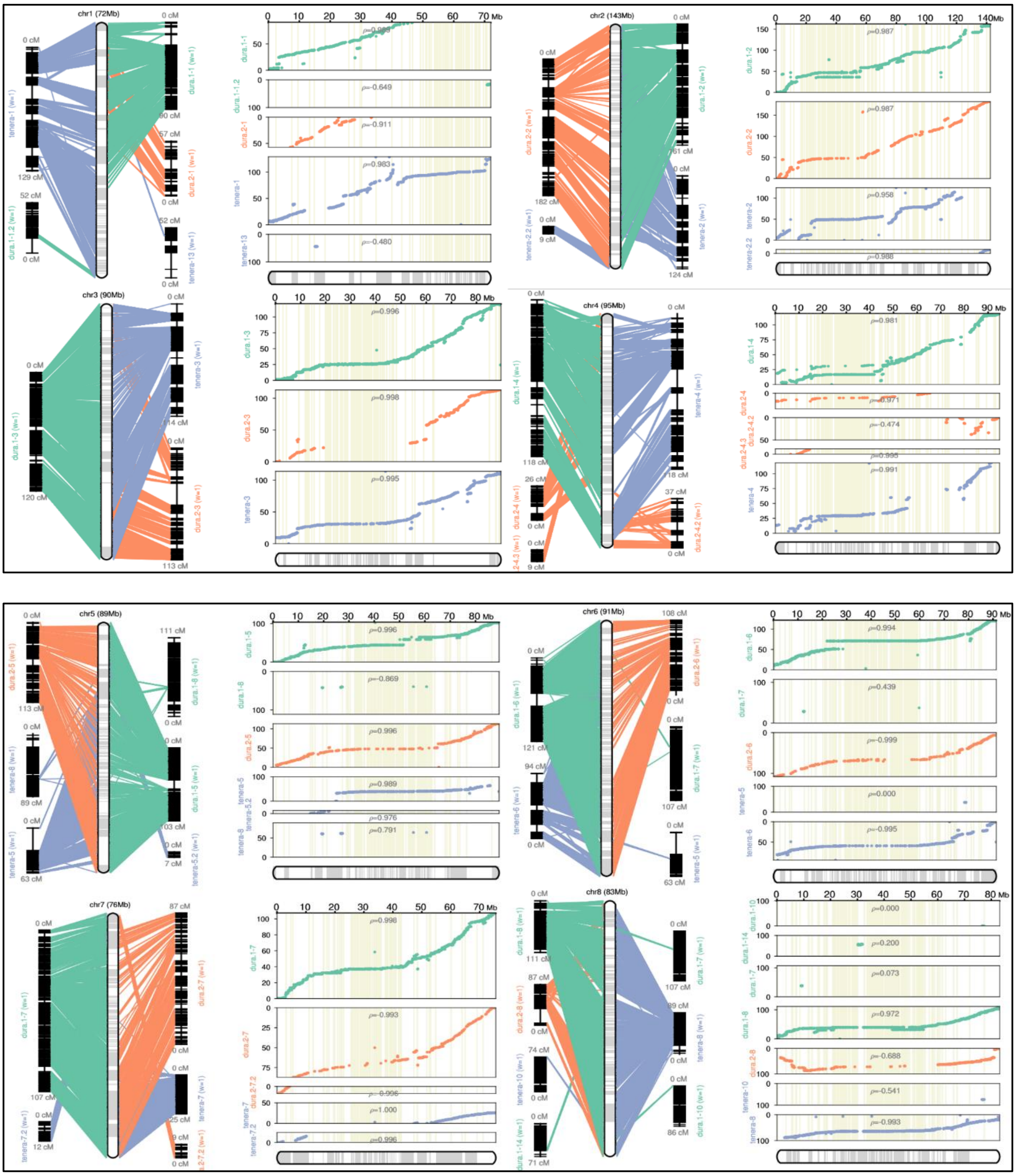

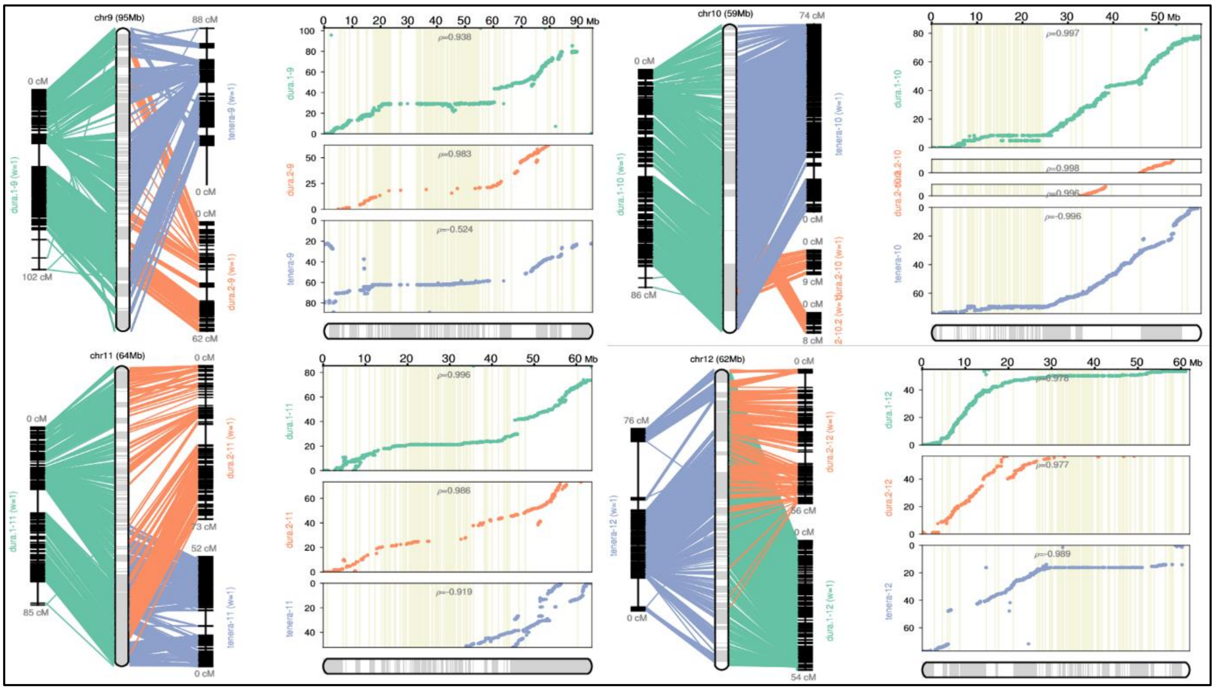
chr9 (95Mb)
dura.1-9 (w=1)
tenera-9 (w=1)
dura.2-9 (w=1)
ρ=0.938
ρ=0.983
ρ=-0.524
chr10 (59Mb)
dura.1-10 (w=1)
tenera-10 (w=1)
ρ=0.997
ρ=0.998
ρ=-0.996
chr11 (64Mb)
dura.1-11 (w=1)
dura.2-11 (w=1)
tenera-11 (w=1)
ρ=0.996
ρ=0.986
ρ=-0.919
chr12 (62Mb)
tenera-12 (w=1)
dura.2-12 (w=1)
dura.1-12 (w=1)
ρ=0.978
ρ=0.977
ρ=-0.989

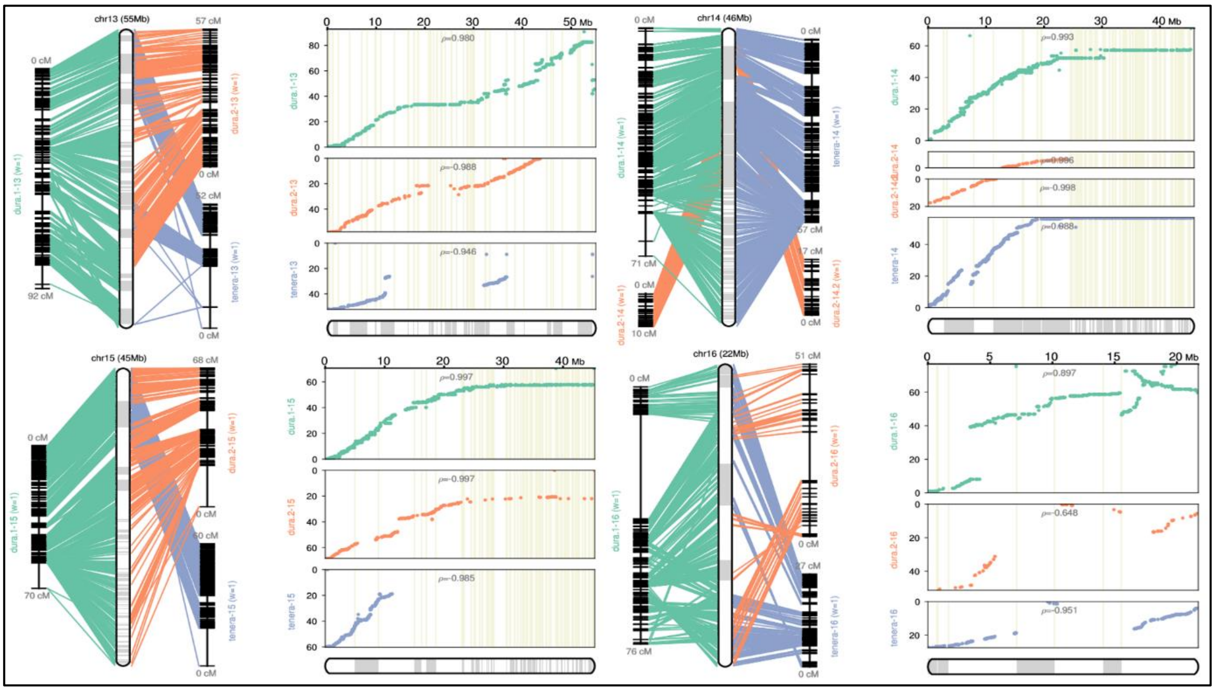
chr13 (55Mb)
dura.1-13 (w=1)
dura.2-13 (w=1)
tenera-13 (w=1)
ρ=0.980
ρ=-0.988
ρ=-0.946
chr14 (46Mb)
dura.1-14 (w=1)
tenera-14 (w=1)
dura.2-14.2 (w=1)
ρ=0.993
ρ=-0.998
chr15 (45Mb)
dura.1-15 (w=1)
dura.2-15 (w=1)
tenera-15 (w=1)
ρ=0.997
ρ=-0.997
ρ=-0.985
chr16 (22Mb)
dura.1-16 (w=1)
dura.2-16 (w=1)
tenera-16 (w=1)
ρ=0.897
ρ=-0.648
ρ=-0.951

Figure S2. Correlation plots for locations of DNA marker located within date palm chromosome (pd-15) for genome assembly improvement versions by Al-Dous,E.K et al. (2011) and Hazzouri, K. M. et al (2019).

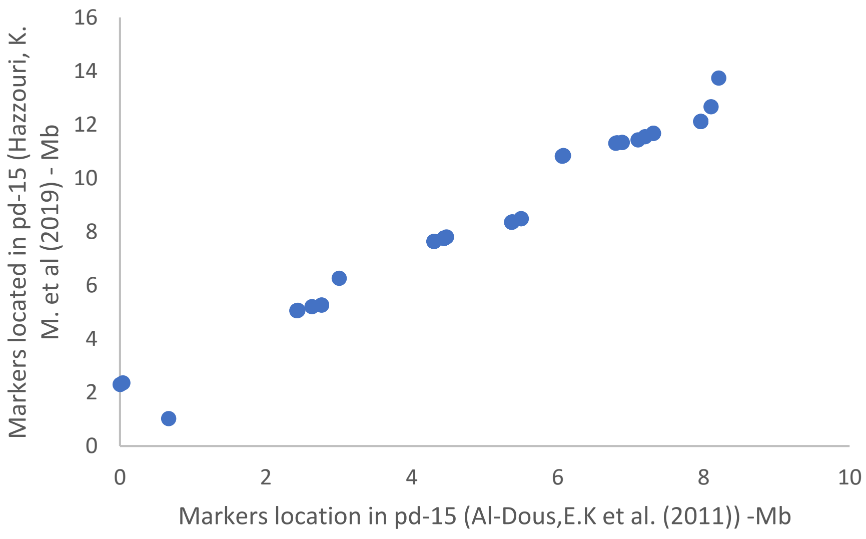

Table S1. Summary for consensus map generated from multiple linkage maps and statistics for SNP markers and scaffolds used to build genome assembly for PMv6.

Summary for Consensus Map			
	Anchored	Oriented	Unplaced
Markers(unique)	39,616	31,657	347
Markers per Mb	33.6	41.7	11.4
Scaffolds	2,272	434	148
Scaffolds with 1 marker	869	0	99
Scaffolds with 2 markers	335	56	24
Scaffolds with 3 markers	175	24	7
Scaffolds with >=4 markers	893	354	18
Total bases	1,178,731,551	759,750,341	30,463,349
Percentage	97.5%	62.8%	2.5%
